# Evaluation of the Sublingual Route for Administration of Influenza H5N1 Virosomes in Combination with the Bacterial Second Messenger c-di-GMP

**DOI:** 10.1371/journal.pone.0026973

**Published:** 2011-11-01

**Authors:** Gabriel Kristian Pedersen, Thomas Ebensen, Ingrid Hjetland Gjeraker, Signe Svindland, Geir Bredholt, Carlos Alberto Guzmán, Rebecca Jane Cox

**Affiliations:** 1 The Gade Institute, University of Bergen, Norway; 2 Department of Vaccinology and Applied Microbiology, Helmholtz Zentrum für Infektionsforschung (HZI), Braunschweig, Germany; 3 Department of Research and Development, Haukeland University Hospital, Bergen, Norway; Centers for Disease Control and Prevention, United States of America

## Abstract

Avian influenza A H5N1 is a virus with pandemic potential. Mucosal vaccines are attractive as they have the potential to block viruses at the site of entry, thereby preventing both disease and further transmission. The intranasal route is safe for the administration of seasonal live-attenuated influenza vaccines, but may be less suitable for administration of pandemic vaccines. Research into novel mucosal routes is therefore needed. In this study, a murine model was used to compare sublingual administration with intranasal and intramuscular administration of influenza H5N1 virosomes (2 µg haemagglutinin; HA) in combination with the mucosal adjuvant (3′,5′)-cyclic dimeric guanylic acid (c-di-GMP). We found that sublingual immunisation effectively induced local and systemic H5N1-specific humoral and cellular immune responses but that the magnitude of response was lower than after intranasal administration. However, both the mucosal routes were superior to intramuscular immunisation for induction of local humoral and systemic cellular immune responses including high frequencies of splenic H5N1-specific multifunctional (IL-2^+^TNF-α^+^) CD4^+^ T cells. The c-di-GMP adjuvanted vaccine elicited systemic haemagglutination inhibition (HI) antibody responses (geometric mean titres ≥40) both when administered sublingually, intranasally and inramuscularly. In addition, salivary HI antibodies were elicited by mucosal, but not intramuscular vaccination. We conclude that the sublingual route is an attractive alternative for administration of pandemic influenza vaccines.

## Introduction

The avian influenza H5N1 continues to cause zoonosis and has the potential to cause the next pandemic. An effective H5N1 vaccine is therefore needed. In contrast to parenteral vaccines, mucosal immunisation can provide local mucosal immunity, which has the potential to prevent influenza infection at the portal of entry [1], [2]. This response is largely mediated by secretory immunoglobulin (Ig) A (sIgA), which is able to neutralise pathogens (Reviewed in [3]). It has also been shown that sIgA antibodies are more cross-reactive towards different strains of influenza than IgG [4], [5]. Furthermore, mucosal vaccines overcome the use of needles, and are thus attractive for use in developing countries. The intranasal (IN) route has been extensively studied [6], [7], [8], [9], [10] and is safely used for the administration of seasonal live-attenuated influenza vaccines in humans (Reviewed in [11]). In contrast, IN vaccination with *Escherichia coli* heat-labile toxin (LT) adjuvanted influenza virosomes significantly increased the risk of Bell's palsy [12]. Later it was discovered that this was probably due to the adjuvant, as another IN formulation (not virosomes) formulated with an LT-derived molecule was also associated with Bell's palsy [13]. Furthermore, IN vaccination has been shown to redirect vaccine antigen and adjuvant components to the central nervous system (CNS) of mice [14], [15], [16]. These findings have prompted exploration of alternative mucosal vaccine routes, particularly for administration of adjuvanted influenza vaccines. The sublingual (SL) route has been used for decades to treat angina [17] and has more recently been investigated for allergen desensitisation therapy [18], [19] and administration of vaccines against various bacterial and viral diseases [20], [21], [22], [23]. An adjuvanted seasonal influenza H1N1 vaccine (whole inactivated A/PR/8) has also proved effective when administered sublingually to mice [15]. Since exposure to H1N1 viruses occurs continually, H1N1 vaccines rarely require adjuvantation to elicit protective immunity. In contrast, efficacious adjuvants are needed to protect the unprimed population against novel influenza subtypes.

In this study we therefore aimed to evaluate the SL route for vaccination against potentially pandemic influenza strains such as avian influenza H5N1. In addition, we compared the immune responses following SL vaccination with the normal routes for influenza vaccines (intramuscular (IM) and IN). We found that SL vaccination of mice with H5N1 virosomes induces both local and systemic humoral and cellular immune responses. Furthermore, by combining the virosomes with a promising mucosal adjuvant, the bacterial second messenger c-di-GMP, the SL vaccine response was boosted even further, as illustrated by high frequencies of spleen-derived multifunctional (IL-2^+^TNF-α^+^) CD4^+^ T cells in addition to seroprotective (geometric mean titres ≥40) haemagglutination inhibition (HI) antibody responses. In contrast to the IM route, both IN and SL administration induced local IgA antibodies and a salivary HI response, which could potentially neutralise influenza virus at the portal of entry. These results support further investigation of the SL route for administration of vaccines against potentially pandemic influenza strains and suggest that c-di-nucleotides might be attractive candidate adjuvants for developing mucosal influenza vaccines.

## Materials and Methods

### Vaccine and Adjuvant

Inactivated influenza virosomal vaccine (Crucell, the Netherlands) was produced as previously described [24], using the reverse genetics seed virus (NIBRG-14), which was derived from a reassortment between A/Vietnam/1194/2004 (H5N1) and A/Puerto Rico/8/34 (H1N1) (The National Institute for Biological Standards and Control (NIBSC), UK) [25]. The virosomes contain the surface haemagglutinin (HA) and neuraminidase proteins embedded in a lipid membrane with no internal proteins. The c-di-GMP adjuvant was synthesized [26], [27] and purified as described previously [10].

### Mice

Female BALB/c mice (six to eight weeks old) were purchased from Charles River laboratories (Sulzfeld, Germany) and housed according to Norwegian National regulations. The study was approved by the Norwegian Animal Research Authority (FDU, ID: 20102742) and conducted according to the Norwegian Animal welfare Act.

### Vaccination and sampling

**Figure 1:**
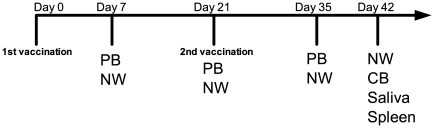
Vaccination scheme and sampling overview. BALB/c mice received two doses (2 µg HA) three weeks apart (Days 0 and 21) of virosomal influenza A/Vietnam/1194/2004 (H5N1) NIBRG-14 vaccine intramuscularly (IM), sublingually (SL) or intranasally (IN) with or without c-di-GMP adjuvant (7.5 µg). An additional group (mock) received PBS and c-di-GMP intranasally. Peripheral blood (PB) and nasal washes (NW) were collected at days 7, 21 and 35 post first immunisation. NW, cardiac blood (CB), saliva and spleens were collected on the day of sacrifice (Day 42).

Mice were divided into groups of six animals and vaccinated IN, IM or SL with two doses (21 days apart) of virosomal influenza A H5N1 NIBRG-14 vaccine (2 µg of HA) with (+) or without (−) c-di-GMP (7.5 µg) adjuvant. Additionally, one group (mock) consisted of six mice which received only the c-di-GMP adjuvant (7.5 µg) by the IN route. IM vaccination was performed by injecting 50 µl into the quadriceps muscles of the hind leg. The mucosally vaccinated mice were deeply anaesthetised by subcutaneous (s.c.) administration of 160 µl of a ketamine (10 mg/ml) and xylaxine (1 mg/ml) mixture. For IN vaccination, the mice were placed in supine position and 3.5 µl of vaccine was administered drop-wise to each nostril. SL vaccination was conducted as described previously [15] by holding the mice in a vertical head-up position and administering 7 µl of vaccine under the tongue. Subsequently, the mice were immediately placed in anteflexion for at least 20 minutes to prevent swallowing of the vaccine. An overview of vaccination and sampling schedules is found in figure 1.

To measure local and systemic influenza-specific antibody responses, blood and nasal washes (flushing twice with 350 µl phosphate buffered saline (PBS)+0.05% bovine serum albumin) were collected on days 7, 21, 35 and 42. In addition, saliva was collected on day 42 by intraperitoneal injection of pilocarpine (0.8 µg/g) to anaesthetised mice (120 µl of a ketamine (10 mg/ml) and xylaxine (1 mg/ml) mixture s.c.). Mononuclear cells were isolated from spleens using Lymphoprep™ (Axis-Shield, Oslo) as previously described [28] and resuspended in lymphocyte medium (RPMI 1640 containing L-glutamine and supplemented with 0.1 mM non-essential amino acids, 10 mM Hepes pH 7.4, 1 mM sodium pyruvate, 50 µM ß-mercapto ethanol, 100 IU/ml penicillin, 100 µg/ml streptomycin, 0.25 µg/ml fungizone and 10% foetal calf serum (FCS)) before use in the cytokine detection and flow cytometry assays.

### Influenza-specific antibodies

The influenza-specific serum, nasal wash and salivary IgA and IgG in addition to serum IgG1 and IgG2a antibodies were quantified using an ELISA assay, as previously described [29], [30] by coating with the NIBRG-14 H5N1 virosomes or Pandemic H1N1 (pH1N1) 2009 whole virus (A/California/7/2009) (2 µg/ml) (kindly provided by NIBSC, UK). The influenza-specific antibody concentrations were calculated using IgA, IgG, IgG1 and IgG2a standards and linear regression of the log-transformed readings.

### Haemagglutination inhibition

Sera and saliva from day 42 were tested for H5N1 (NIBRG-14) HI antibodies by standard methods using a 0.7% v/v turkey erythrocyte suspension. To remove non-specific inhibitors, sera and saliva were treated 1∶5 and 1∶2, respectively with receptor-destroying enzyme (RDE; Seiken, Japan) overnight, before heat-inactivation (56°C, 30 min). Sera and saliva samples were added to 96-well v-bottomed microtiter plates at a starting dilution of 1∶10 and 1∶4 respectively. The serum and saliva HI titres are expressed as the reciprocal of the highest dilution at which 50% haemagglutination was inhibited. A surrogate correlate of protection was extrapolated from seasonal vaccination in humans, using a titre ≥40 to indicate seroprotection [31] and negatives were assigned a titre of 5 and 2 for serum and saliva, respectively.

### Cytokine detection

Cytokine secretion was investigated on day 42. To this end, mononuclear cells from spleens (10^6^ per well) were incubated (37°C, 5% CO_2_) for 72 hours in 200 µl of lymphocyte medium containing 2.5 µg HA/ml of virosomal influenza NIBRG-14 H5N1 antigen or medium alone. After incubation, the supernatants were stored at −80°C until used. The Bio-plex (Bio-rad, USA) cytokine kits were used according to the manufacturer's instructions to quantify cytokines of the T helper 1 (Th1) (IFN-γ, IL-2), Th2 (IL-4, IL-5 and IL-10) and Th17 (IL-17) subsets. The cytokine concentrations for each individual mouse were calculated by subtracting the basal release (concentrations in supernatants from cells incubated with lymphocyte medium alone) from the concentrations in supernatants of cells stimulated with H5N1 influenza antigen. The cut-off point in the assay was 10 pg/ml.

### CD4^+^ Th1 cell responses

Mononuclear cells (10^6^ cells per well) from spleens were incubated (37°C, 5% CO_2_) overnight in 200 µl lymphocyte medium containing 2.5 µg/ml HA of virosomal influenza NIBRG-14 H5N1 antigen (Crucell, The Netherlands) or pandemic H1N1 (pH1N1) A/California/7/2009-like split virus (X179a, GlaxoSmithKline, Belgium), 2 µg/ml anti-CD28 (Pharmingen, USA) and 10 µg/ml Brefeldin A (BD Biosciences, USA). The basal cytokine production was determined by incubating splenocytes from vaccinated mice in the same medium but without antigen and the percentages of cytokine positive cells were subtracted from the influenza-stimulated cells. As positive controls, cells were incubated in medium containing the mitogens phorbol myristate acetate (10 ng/ml) and ionomycin (250 ng/ml). Subsequently, cells were stained for CD3, CD4, CD8, IFN-γ, IL-2 and TNF-α (BD Biosciences, USA) using the BD Cytofix/Cytoperm kit according to the manufacturer's instructions and as previously described [32]. The cells were resuspended in PBS containing 5% FCS and 0.1% sodium azide and light emission was measured by BD FACSCanto flow cytometer (acquiring at least 3×10^5^ cells per sample). Data were analyzed using FlowJo v8.8.6 (Tree Star, USA), Pestle v1.6.2 and SPICE v5.1 (Mario Roederer, Vaccine Research Centre, NIH, USA) and multifunctional CD4^+^ Th1 cells were identified as previously described [30], [32], [33]. T cells were classified based on cytokine IFN-γ, IL-2 and TNF-α secretion as single producers (one cytokine), double producers (two cytokines) and triple producers (all three cytokines). In addition, the percentages of CD4^+^ cells producing non-overlapping permutations of the analyzed cytokines were summed to quantify each mouse's total frequency of influenza specific CD4^+^ Th1 cells.

### Memory B cells

Memory B cells were detected as described previously [34] with the following modifications. Isolated splenic mononuclear cells were seeded in 24-well plates at 5×10^5^ cells/well in 1 ml lymphocyte medium containing 0.1 µg/ml pokeweed mitogen (Sigma-Aldrich, USA) and 0.001% heat-killed, formalin-fixed Staphylococcus aureus Cowan I strain (Merck Chemicals, USA) for 6 days at 37°C, 5% CO_2_. Splenocytes were incubated likewise in the absence of mitogens to substantiate the detection of memory B cells and not plasma cells. ELISPOT plates (MSHAN45, Millipore, USA) were coated with 2 µg/ml of the NIBRG-14 H5N1 virosomes or 2 µg/ml of goat anti-mouse IgG (Southern Biotech, USA) in sterile PBS and 2.5×10^5^ cells/ml lymphocyte medium were added and incubated for 16 hours at 37°C, 5% CO_2_. The plates were developed with biotin-conjugated goat anti-mouse IgG (Southern Biotech), extravidin peroxidase (Sigma-Aldrich) and TMB-H peroxidase (Moss, inc., USA). The spots were counted using an Immunoscan™ reader (CTL-Europe, Germany) and spots in wells with non-stimulated cells were subtracted from corresponding wells with mitogen-stimulated cells. The data show the percentage of H5N1-specific memory B-cells of the total IgG producing memory B-cell response as suggested previously [34].

### Proliferation

Poliferation was measured by thymidine incorporation in splenocytes isolated three weeks after the second vaccine dose. Cells were diluted (10^7^ cells/ml) in lymphocyte medium and stimulated in 96-well plates with 2.5 µg/ml of virosomal H5N1 antigen. As a positive control, cells were stimulated with the T-cell mitogens phorbol myristate acetate (10 ng/ml) and ionomycin (250 ng/ml). A negative control of cells stimulated with lymphocyte medium alone was subtracted from influenza-stimulated cells. After 72 hours, 1 µCi ^3^H-thymidine in 25 µl of medium per well was added and the plates were incubated for 16 hours. Subsequently the cells were harvested, 10 µl scintillation fluid added and the incorporation of ^3^H thymidine determined by scintillation spectroscopy.

### Statistical analysis

To evaluate the potential of SL administration for delivery of pandemic influenza vaccines, we used the IN and IM routes as golden standards and compared the local and systemic humoral and cellular immune responses following administration by each route. Differences between groups were analyzed by one-way ANOVA with Bonferroni's adjustment (GraphPad Prism v5.0d for Mac). Additionally, a student T-test integrated in SPICE v5.1 [35] was used for the comparison of CD4^+^ T-cell frequencies. Analysis and presentation of T-cell distributions was performed using SPICE version 5.1, downloaded from http://exon.niaid.nih.gov/spice [35].

## Results

### Sublingual H5N1 vaccination induces local and systemic antibody responses

**Figure 2:**
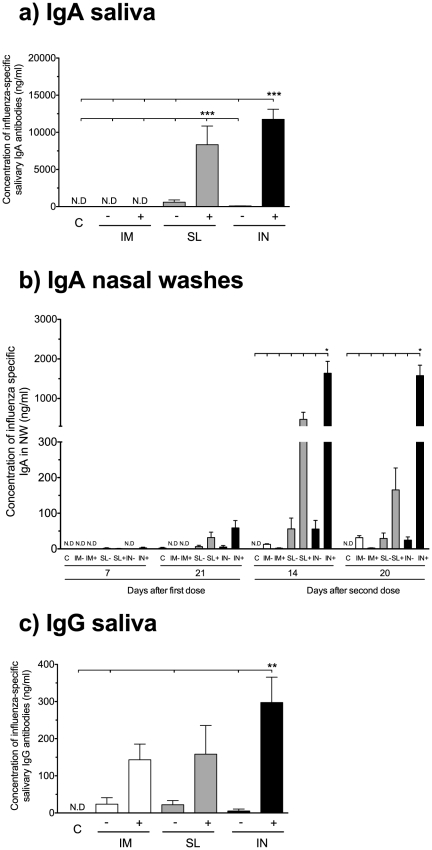
Only SL and IN vaccination induces IgA in the local mucosa. The local influenza-specific IgA antibody concentrations were measured in the saliva and nasal washes. **a**) The concentrations of IgA in the saliva three weeks after the second immunisation. **b**) The kinetics of the IgA responses in the nasal washes (NW). **c**) The local influenza-specific IgG concentrations in saliva. Each bar represents mean antibody concentration + SEM. *, ** and *** indicate statistically significant differences between groups (p<0.05, p<0.01 and p<0.001 respectively, One-way ANOVA with Bonferroni's correction for multiple group comparison). N.D = not detected. BALB/c mice were vaccinated intramuscularly (IM), sublingually (SL) or intranasally (IN) with (+) or without (−) c-di-GMP adjuvant. An additional group received a mock vaccine (C) of c-di-GMP alone administered IN.

Mucosal vaccines should induce both local and systemic antibody responses. We thus determined the influenza-specific IgA and IgG antibody concentrations in local secretions and serum after vaccination. The highest salivary IgA concentrations were found in the groups receiving the adjuvanted vaccine and the influenza-specific IgA concentrations were significantly higher in the intranasal c-di-GMP adjuvanted (IN^+^) and sublingual c-di-GMP adjuvanted (SL^+^) groups than in all other groups (p<0.001). In contrast, substantially lower concentrations of IgA were observed in the non-adjuvanted mucosal groups and no IgA could be measured in saliva from mice receiving the IM vaccines (figure 2a). The c-di-GMP adjuvanted mucosal vaccines also induced the highest influenza-specific IgA concentrations in the nasal washes (figure 2b) with the highest concentrations observed in the IN^+^ group (significantly higher than in all other groups at 14 and 21 days after the second dose, p<0.05) followed by the SL^+^ group. We also measured the influenza-specific pIgR concentrations in nasal washes and saliva by ELISA to confirm that the IgA was locally produced (sIgA) rather than a transudate from serum. None of the groups vaccinated with the virosomes alone or the intramuscular c-di-GMP adjuvanted (IM^+^) group had detectable pIgR levels in nasal washes. In contrast, pIgR was detected in both the SL^+^ and IN^+^ groups and a significant correlation between IgA and pIgR concentrations was observed (data not shown). Influenza-specific salivary IgG was detected in all vaccinated groups and the highest concentrations were observed in the IN^+^ group (significantly higher than the non-adjuvanted groups, p<0.01), followed by the SL^+^ and IM^+^ groups (figure 2c). The c-di-GMP adjuvant enhanced the salivary IgG responses irrespective of the route of administration as observed by higher mean concentrations (>140 ng/ml) in the IM^+^, SL^+^ and IN^+^ groups as compared to their non-adjuvanted counterparts (<20 ng/ml).

**Figure 3:**
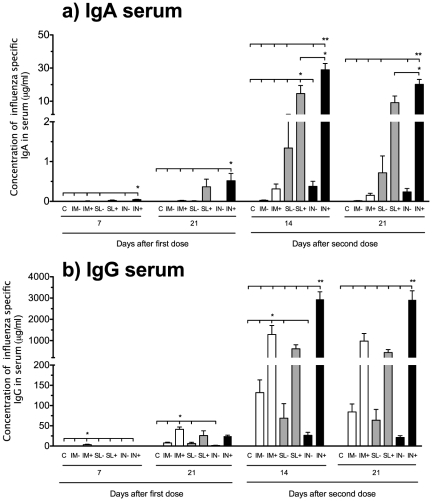
Systemic antibody kinetics following immunization. The **a**) IgA and **b**) IgG serum influenza-specific antibody concentrations were measured at 7 and 21 days after the first vaccine dose and 14 and 21 days after the second dose. Each bar represents mean antibody concentration + SEM. * and ** indicate statistically significant differences between groups (p<0.05 and p<0.01 respectively, One-way ANOVA with Bonferroni's correction for multiple group comparison). Groups of six mice were vaccinated intramuscularly (IM), sublingually (SL) or intranasally (IN) with a virosomal H5N1 vaccine (NIBRG-14) with (+) or without (−) c-di-GMP adjuvant. An additional group received a mock vaccine (C) of c-di-GMP alone administered IN.

The highest systemic IgA responses were also found in the c-di-GMP adjuvanted mucosal vaccine groups. Thus, significantly higher IgA concentrations were found in the IN^+^ (p<0.05 throughout the study) and SL^+^ groups (p<0.05 at 14 days after the second dose) as compared to all other groups (figure 3a). Systemic IgA responses were observed already at day seven after the first dose in the SL^+^ (23 ng/ml) and IN^+^ (45 ng/ml) groups. In contrast, the highest serum IgG concentration (3.5 µg/ml) was seen in the IM^+^ group at seven days after the first immunisation (significantly higher than all other groups, p<0.05) (figure 3b). However, after the second dose the IN^+^ group showed the highest serum IgG response, being significantly higher (p<0.01) than in all other groups. We then continued to analyse the capability of the vaccine to induce cross-reactive antibody responses towards a heterosubtypic influenza strain, pH1N1 (figure S1). Only low concentrations of pH1N1-specific antibodies were observed when the virosomes were administered alone. However, the c-di-GMP adjuvanted vaccine induced systemic pH1N1-specific IgG responses by all three routes (figure S1a). In addition, local pH1N1-specific IgA responses were induced when the vaccine was administered mucosally (figure S1b) and the highest concentrations were observed when the vaccine was given IN (significantly higher than all but the SL^+^ group, p<0.001).

### Local and systemic antibodies capable of haemagglutination inhibition are induced by sublingual H5N1 vaccination

**Figure 4:**
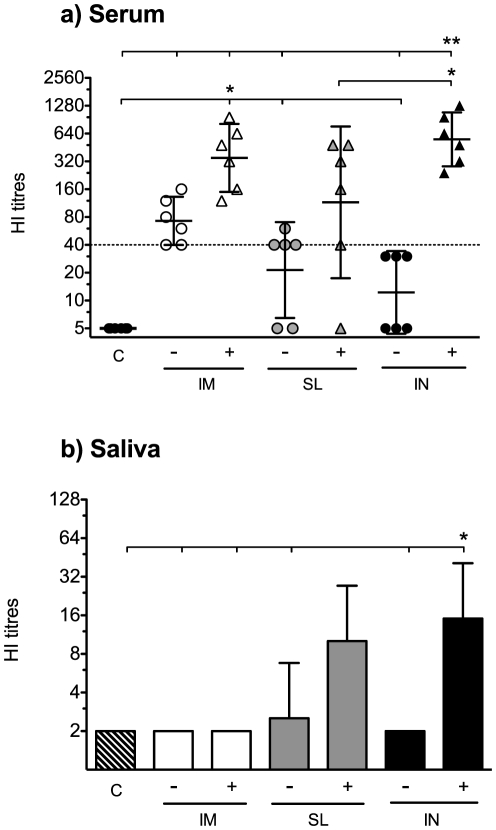
The c-di-GMP adjuvanted vaccine induced both systemic and local haemagglutination inhibition responses when administered mucosally. Haemagglutination inhibition (HI) antibodies were measured at day 42 in a) serum. The data show the response of each individual mouse and the geometric mean titres ±95% confidence interval. The dotted line represents an HI titre of 40. b) The local HI response was measured in saliva at day 42 after intraperitoneal injection of pilocarpine and collection of saliva. To obtain enough sample volume, the salivary HI assay was conducted on pooled saliva samples. The data show the geometric mean titres +95% confidence interval of three independent experiments. * and ** indicate statistically significant differences between groups (p<0.05 and 0.01 respectively, One-way ANOVA with Bonferroni's correction for multiple group comparison). The mice (six per group) were vaccinated with the virosomal H5N1 vaccine (NIBRG-14) alone (−) or in combination with (+) c-di-GMP adjuvant by the intramuscular (IM), sublingual (SL) or intranasal (IN) route. One additional group received a mock vaccine (C) of c-di-GMP administered alone by the IN route.

Since the virosomal vaccine induced antibody responses when administered by the SL route, we continued to evaluate the functionality of the local and systemic antibodies in the HI assay. Sera isolated from cardiac blood at day 42 were tested for HI antibodies against the homologous NIBRG-14 strain. HI titers ≥40 were regarded as indicative of protection, although this surrogate correlate of protection is only established for seasonal influenza vaccines in man. Groups vaccinated with virosomes alone had lower geometric mean titres (GMT) than their respective adjuvanted groups but in the IM- group, all mice had HI-titres ≥40 (GMT = 70). The c-di-GMP adjuvant enhanced the HI response, and all adjuvanted groups obtained HI GMT≥40 by all administration routes (figure 4a). However, one mouse in the SL^+^ group did not obtain an HI antibody response and this mouse also responded poorly, particularly in the other antibody assays. Comparing the different routes of administration, it was found that the IN^+^ group had the highest HI titres (GMT = 550), followed by the IM^+^ (GMT = 350) and the SL^+^ (GMT = 115) groups. Thus, when the virosomes were given alone, only the IM route elicited seroprotective HI titres, whilst the virosomes in combination with c-di-GMP adjuvant induced protective HI GMT by all the evaluated routes of administration.

As a measure of functionality of the local mucosal antibody response, we tested saliva samples for HI antibodies against the vaccine strain (NIBRG-14). The IN^+^ group had the highest HI GMT (GMT = 15) in saliva followed by the SL^+^ group (GMT = 10) (figure 4b). Among the non-adjuvanted groups, only the SL^−^ group had a response (GMT = 3), whereas no salivary HI antibody responses were observed in the intramuscular or control groups.

### Sublingual H5N1 vaccination with c-di-GMP adjuavnted virosomes induces a balanced Th1/2 profile and Th17 responses

**Figure 5:**
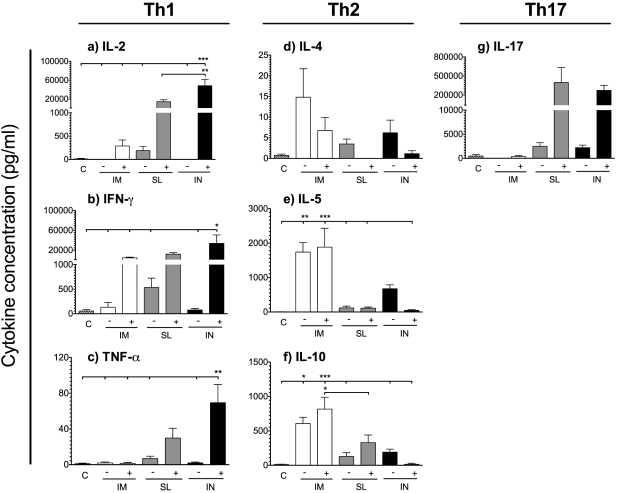
The adjuvanted SL and IN vaccines induced the highest Th1 and Th17 cytokine concentrations. BALB/c mice were vaccinated intramuscularly (IM), sublingually (SL) or intranasally (IN) with virosomal H5N1 vaccine (NIBRG-14) alone (−) or with (+) c-di-GMP adjuvant. An additional group received a mock vaccine of c-di-GMP administered alone IN (C). Splenocytes were isolated three weeks after the second immunisation and incubated for 72 hours with 2.5 µg/ml of H5N1 virosomal NIBRG-14 before analysis by Bio-plex for **a**) IL-2, **b**) IFN-γ, **c**) TNF-α, **d**) IL-4, **e**) IL-5, **f**) IL-10 and **g**) IL-17. The cytokine concentrations for each mouse were calculated by subtracting the basal release of unstimulated samples from that of stimulated samples. Each bar represents mean values from six mice and error bars indicate SEM. *, ** and *** indicate statistically significant differences between groups (p<0.05, p<0.01 and p<0.001 respectively, One-way ANOVA with Bonferroni's correction for multiple group comparison).

Pandemic influenza vaccines should preferentially activate both Th1 (IL-2, IFN-γ and TNF-α) and Th2 subsets (IL-4, IL-5, and IL-10) of T helper cells, since both are important for elimination of influenza virus from the host [36]. To examine the effect of SL vaccination on the polarisation of CD4^+^ T-cell responses, mononuclear cells from spleens were stimulated *ex vivo* for 72 hours with the NIBRG-14 virosomes and the supernatant was analysed for production of cytokines. Generally, low levels of Th1 cytokines were produced in the non-adjuvanted groups, whilst adjuvantation with c-di-GMP markedly enhanced the cytokine responses (figure 5a, b and c). Thus, all the adjuvanted groups produced IL-2, whilst the only IL-2 producing non-adjuvanted group was the SL^−^ group (figure 5a). Similarly, high concentrations of IFN-γ were produced in the SL^+^ (mean of 12 ng/ml), IN^+^ (mean of 34 ng/ml) and IM^+^ (mean of 5 ng/ml) groups as compared to the non-adjuvanted groups (mean <0.6 ng/ml for all groups) (figure 5b). When comparing the routes of administration, we found that the mucosal routes (IN and SL) induced a higher production of all the Th1 cytokines measured than the IM route. In contrast, low levels of the Th2 cytokines IL-4, IL-5 and IL-10 were found in the IN^+^ and SL^+^ groups as compared to the IM- and and IM^+^ groups (figure 5d, e and f). Since we have previously found that IN influenza vaccination induces IL-17 [37], [38], we analysed if IL-17 would also be produced by SL vaccination. Indeed, IL-17 was produced by both SL and IN vaccinated mice, whilst IM vaccination did not induce an IL-17 response.

**Figure 6:**
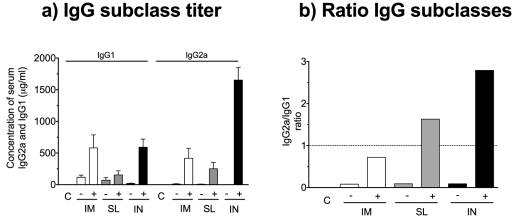
The c-di-GMP adjuvant altered the IgG2a/IgG1 ratio towards a more Th1 skewed phenotype. Intramuscular (IM), sublingual (SL) and intranasal (IN) administration of BALB/c mice was performed with the virosomal H5N1 vaccine (NIBRG-14) in combination with (+) c-di-GMP adjuvant or alone (−). An additional group received a mock vaccine (C) with only c-di-GMP administered IN. **a**) The concentration of IgG1 and IgG2a in the serum at two weeks after the second immunisation. Each bar represents mean antibody concentration + SEM. **b**) The IgG2a/IgG1 ratio (ratios>1 and <1 indicate a Th1 and Th2 polarised response, respectively).

To further substantiate the differences in Th profiles observed between the SL route and the IM and IN routes, we measured the IgG2a/IgG1 ratio in serum collected two weeks after the last of two immunisations. For the IgG subclasses (IgG1 and IgG2a), all the non-adjuvanted groups induced a Th2 skewed response indicated by a predominant production of IgG1 (figure 6a and b). In contrast, inclusion of the c-di-GMP adjuvant enhanced the production of IgG2a antibodies and both the SL^+^ and IN^+^ groups had an IgG2a/IgG1 ratio >1, whereas the IM^+^ group had a ratio<1. Thus, the cytokine profiling and IgG subtype analysis suggest that vaccination with the c-di-GMP adjuvanted virosomes IN and SL elicits a predominant Th1 and Th17 response, whilst IM vaccination with the c-di-GMP adjuvanted virosomes or administration of the virosomes alone induces a Th2 polarisation.

### Mucosal administration of the c-di-GMP adjuvanted virosomes induces high frequencies of homologous (H5N1) and heterologous (pH1N1) influenza-specific CD4^+^Th1 cells

**Figure 7:**
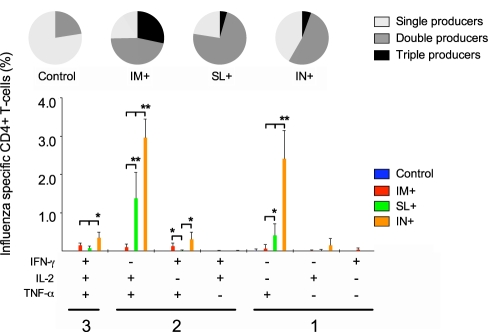
The c-di-GMP adjuvanted vaccine induced the highest frequencies of influenza-specific CD4+ T-cells when given mucosally. The CD4+ T-cell functional responses were measured by stimulating splenocytes *ex vivo* with the H5N1 virosomes before fixation, staining for IFN-γ, IL-2 and TNF-α and analysis by flow cytometry. The bars show the frequencies of CD4^+^ T-cells producing any of the seven possible combinations of the measured cytokines. The pie charts show the fraction of CD4^+^ T-cells within each group producing any one, any combination of two or all three cytokines simultaneously + SD. * and ** indicate statistically significant differences between groups (p<0.05 and p<0.01 respectively measured by the Student t-test). BALB/c mice were vaccinated with c-di-GMP adjuvanted virosomal H5N1 vaccine (NIBRG-14) by the intramuscular (IM+), sublingual (SL+) or intranasal (IN+) route.

CD4^+^ T cells expressing a multifunctional polarisation in the cytokines produced have been shown to be a correlate of protection against bacterial and protozoan diseases [32], [39]. We have previously found that high frequencies of multifunctional CD4^+^ T cells are induced when the NIBRG-14 virosomes are administered in combination with the saponine based Matrix M™ adjuvant [30], [37]. Here we assessed the ability of the c-di-GMP adjuvanted virosomes to induce multifunctional CD4^+^ Th1 cells (concurrently producing two or three of the cytokines IL-2^+^, IFN-γ^+^ and TNF-α^+^) when co-administered by the SL route. To this end, mononuclear cells from spleens were isolated three weeks after the second immunisation and stimulated *in vitro* with the virosomal H5N1 vaccine (NIBRG-14). Subsequently the cells were stained for CD3 and CD4 and intracellularly for IL-2, IFN-γ and TNF-α. Only low levels of H5N1-specific cytokine producing cells were detected in the non-adjuvanted groups and no significant differences were observed between the non-adjuvanted groups (data not shown). In contrast, when the virosomes were combined with c-di-GMP, high frequencies of IL-2, IFN-γ and TNF-α producing cells were induced (figure 7). Overall, the IN^+^ group had the highest frequencies of H5N1-specific cytokine producing CD4^+^ Th1 cells followed by the SL^+^ group (figure 7). Thus, the IN^+^ group had the highest frequencies of CD4^+^ T cells simultaneously producing all three of the measured cytokines (significantly higher than the IM^+^ and SL^+^ groups, p<0.05), whilst no significant differences were found between the IM^+^ and SL^+^ groups in terms of triple cytokine producing cells. CD4^+^ T cells simultaneously producing IL-2 and TNF-α dominated the double cytokine producing cell response and the highest cell frequencies were found in the IN^+^ and SL^+^ groups (both significantly higher than the IM^+^ group, p<0.01). In contrast, a low frequency of cells in the SL^+^ group produced IFN-γ and TNF-α or IFN-γ and IL-2 simultaneously. The single-cytokine producing cells synthesised mainly TNF-α and the highest frequencies of these cells were observed in the IN^+^ (significantly higher than in the IM^+^ and SL^+^ groups, p<0.01) and SL^+^ (significantly higher than the IM^+^ group, p<0.05) groups. The proportions of triple producers, double producers and single producers are shown in the pie charts (figure 7). When focusing on the distribution of the single, double and triple producers, the IM^+^ group had the largest proportion of triple producers with approximately 30% of the CD4^+^ T cells in the IM^+^ group producing all three cytokines. In contrast, the majority of CD4^+^ T cells in the SL^+^ and IN^+^ groups were double producers.

**Figure 8:**
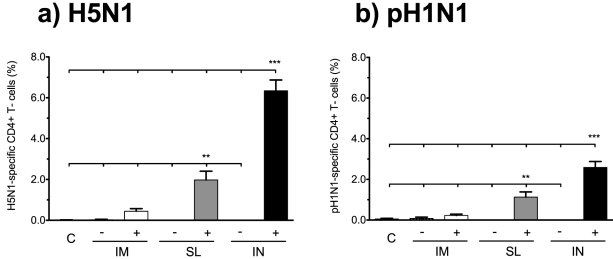
Sublingual and intranasal vaccination induces high frequencies of CD4^+^ T-cells cross-reactive towards pH1N1 virus. The frequencies of influenza specific CD4+ T-cells were measured as described in figure 7 by stimulation with **a**) the H5N1 virosomes and **b**) the pH1N1 A/California/7/2009-like virus (X179a). The bars show total frequencies of influenza-specific CD4^+^ T-cells (the percentages of cells producing any one or more of the measured cytokines) + SEM. ** and *** indicate statistically significant differences between groups (p<0.01 and p<0.001 respectively, One-way ANOVA with Bonferroni's correction for multiple group comparison). BALB/c mice were vaccinated intramuscularly (IM), sublingually (SL) or intranasally (IN) with virosomal H5N1 vaccine (NIBRG-14) alone (−) or in combination with (+) c-di-GMP adjuvant. An additional group received a mock vaccine (C).

It has previously been reported that CD4^+^ T cells can cross-react between influenza virus subtypes [40], [41], [42], [43]. We thus continued to investigate if SL vaccination with the c-di-GMP adjuvanted virosomal vaccine would induce hetero-subtypic cross-reactive CD4^+^ T cell responses, by stimulating with pH1N1 antigen (figure 8). All permutations of the produced cytokines were summed to measure the total frequency of influenza specific CD4^+^ Th1 cells. Similarly to homologous stimulation, pH1N1 stimulation induced the highest frequencies of cytokine producing CD4^+^ T cells in the groups receiving the c-di-GMP adjuvanted vaccine IN (significantly higher than all other groups, p<0.001) and SL (significantly higher than all other groups except the IN^+^, p<0.01) illustrating that hetero-subtypic CD4^+^ Th1 cell responses can be activated by SL administration. Thus, although the c-di-GMP adjuvanted vaccine induced CD4^+^ Th1 cell responses when administered IM, the frequencies of homologous and hetero-subtype influenza-specific CD4^+^ Th1 cells were significantly higher upon IN and SL administration.

## Discussion

Mucosal vaccination against H5N1 virus has many potential benefits, such as limiting transmission, inducing cross-reactive immune responses that could prevent disease caused by drifted strains [4], [5] and being suitable for use in low-income countries. Numerous studies have highlighted the need for an effective adjuvant to elicit seroprotective responses against H5N1 viruses (Summarised in [44]). Although IN vaccination against influenza has proven highly effective, the use of adjuvants poses a problem since adjuvanted IN administered vaccines were associated with the facial nerve disease Bell's palsy [12], [13]. Thus, alternative mucosal routes for H5N1 vaccines are highly desirable. In contrast to the olfactory epithelium, which is in close proximity to the brain, the SL route is anatomically more distant, thus minimising the risk of neurological side effects, whilst providing local mucosal respiratory immunity. In this study we present a head-to-head comparison of the immune responses induced by influenza vaccination through the IM, IN and SL routes and show that H5N1 virosomes in combination with a novel effective mucosal adjuvant can be highly immunogenic when administered by the SL route. Virosomes are potent stimulators of mucosal immunity (Reviewed in [45]) and SL vaccination with the c-di-GMP adjuvanted H5N1 virosomes induced both local and systemic antibody responses and high frequencies of influenza-specific homo- and hetero-subtypic CD4^+^ Th1 cells. These responses appeared qualitatively similar to when the vaccine was administered IN, but were reduced in magnitude. Likely explanations for the differences in magnitude could be that SL administration of vaccine antigen has induced an unintended tolerogenic response, although the low concentration of IL-10 does not support this, or that the vaccine components have been subjected to enzymatic degradation in the saliva before absorption. Another possibility is that the antigen uptake and processing under the tongue is different than of the nose, which is supported by a previous study showing that the nasal associated lymphoid tissues may be a superior mucosal site as compared to other mucosal associated lymphoid tissues [46]. Finally, deglutition of vaccine components cannot be excluded and our studies suggest that c-di-GMP exhibits reduced activity when administered orally (unpublished data). Nonetheless, a low volume (≤7 µl) was administered to minimise this risk [15]. We have previously evaluated a c-di-GMP adjuvanted plant produced H5 HA1 vaccine for IN and IM administration. Compared to this vaccine candidate, IM administration of the virosomes offered significantly higher humoral and cellular responses both when administered alone and in combination with c-di-GMP [47]. However, when comparing the two vaccine candidates for IN administration we found that the responses were of the same order of magnitude, which may suggest that the virosomal formulation offers little advantage in terms of inducing a mucosal immune response and illustrates the importance of including a potent adjuvant in mucosal vaccine formulations. In this context, we found that the STING receptor ligand c-di-GMP [48] boosted the mucosal vaccine responses significantly.

A previous study has shown that SL vaccination with an inactivated whole H1N1 strain elicits protection against lethal viral challenge with influenza [15]. We have not evaluated the protective efficacy of SL vaccination with the c-di-GMP adjuvanted NIBRG-14 H5N1 virosomes, but our previous results showed that two IM doses (1 µg HA) of the NIBRG-14 H5N1 virosomal vaccine alone protected BALB/c mice from lethal viral challenge with highly pathogenic avian influenza (HPAI) H5N1 [37]. In the current study we found that the same vaccine, when combined with c-di-GMP and administered by the SL route, induces stronger humoral and cellular immune responses than the virosomal vaccine alone given IM. Extrapolating from our previous results, we can therefore predict that SL vaccination of the c-di-GMP adjuvanted vaccine would probably also protect mice from challenge with HPAI. This is supported by the finding of protective serum HI antibody responses (HI titres ≥40) in five of six mice the SL group three weeks after the second immunisation (figure 4a). Furthermore, SL vaccination with the c-di-GMP adjuvanted vaccine induced salivary HI antibodies as opposed to the IM route (figure 4b), although it remains unclear if these levels of locally secreted antibodies would be sufficient to prevent transmission of influenza [49]. Notably, a salivary surrogate correlate of protection would likely be lower than in the serum (HI titre ≥40), because salivary antibodies should only be able to overcome the initial viral load, whilst serum antibodies should prevent disease despite viral replication in the respiratory tract.

The c-di-GMP adjuvanted vaccine induced local influenza-specific IgA in the SL and IN groups, but not in the IM groups, which confirms that parenteral vaccination induces only limited mucosal IgA antibody-responses [1]. To substantiate that the IgA antibodies were locally produced rather than derived by transudation, we measured the H5N1-specific pIgR responses in salivary secretions and found that only the mucosal routes elicited pIgR production. In addition, we found that the concentrations of IgA reflected those of pIgR (data not shown) as has been reported earlier [50]. Interestingly, H5N1-specific IgG was detected in saliva following vaccination by all routes, but it is unclear if these antibodies are locally produced or if they originate from serum. Of note, despite having IgG antibodies in saliva, a salivary HI response was not measured in the IM^+^ group and we thus propose that the secretory HI response is due to locally produced IgA. The systemic IgG levels were highest in the IN^+^ group, whilst no differences in IgG concentrations were observed between the IM^+^ and SL^+^ groups. To get an indication of the long-term effectiveness of the vaccine, we measured the frequency of memory B cells, by mitogenic stimulation of splenocytes for 6 days and subsequent ELISPOT for detection of H5N1-specific IgG producing cells [34], [51], [52] and found that the mucosal vaccines induced a superior memory B cell response to intramuscular vaccination (figure S2a).

Influenza infection induces a predominant Th1 response [53], [54]. In contrast, inactivated influenza vaccines have been reported to stimulate Th2-skewed responses [37], [55], [56]. Here we show that the H5N1 virosomes alone induced a Th2 response upon mucosal (SL and IN) administration (figure 6), in accordance with our previous findings [30], [37]. However, the inclusion of c-di-GMP adjuvant resulted in a skewing of the response towards a balanced or predominant Th1 response. This is an important property of the c-di-GMP adjuvant [10], [57], since Th1 cells are important for recovery from influenza viral infection [36], [58]. Furthermore, a Th2 response can potentially be detrimental due to development of asthma (reviewed in [59]). IL-17, the Th17 cytokine, is elicited by IN vaccination [37], [38]. It has also been shown that SL vaccination with a bacterial antigen can induce IL-17 [21]. Here we extend these results to include SL vaccination against influenza and we speculate if IL-17 can be used as a mucosal immune response signature. However, the importance of IL-17 for influenza disease outcome remains controversial. In one study, IL-17RA knockout mice had improved survival following lethal influenza viral challenge as compared to wild type mice [60], suggesting a detrimental effect of IL-17 in the course of influenza infection. In contrast, another study showed that adoptively transferred Th17 effector cells can protect naïve mice against lethal influenza viral challenge [61] and it was recently shown in a mouse model that IL-17 plays a crucial role for B cell responses to influenza H5N1 infection [62]. We have previously found that IM vaccinated mice were protected against homologous HPAI challenge despite not producing IL-17 upon *ex vivo* re-stimulation of splenocytes [37], which suggests that IL-17 is dispensable in terms of effective vaccination against influenza. More studies are therefore needed to elucidate whether IL-17 has an important function in protection against influenza disease. Interestingly, a recent study showed that IL-17 can contribute to generation of a Th1 response [63], which may at least in part explain why the mucosal groups in our study showed a Th1 skewed response and very high frequencies of H5N1 specific CD4^+^ Th1 cells, whilst IM vaccination induced a Th2 polarised response and lower CD4^+^ Th1 cell frequencies.

The c-di-GMP adjuvanted virosomal H5N1 vaccine induced homologous (H5N1) and hetero-subtypic (pH1N1) influenza-specific CD4^+^ Th1 cells and significantly higher (p<0.01) frequencies of CD4^+^ Th1 cells were produced after mucosal (IN and SL) than IM administration (figure 8). To elucidate if overall higher T cell responses were induced by mucosal (SL or IN) rather than IM administration, we measured H5N1-specific proliferation of mononuclear cells from the spleens three weeks after the second immunisation and found no significant differences in proliferation between these routes of immunisation (figure S2b). Interestingly, the Th1 cell cytokine profile was dominated by double cytokine (IL-2^+^TNF-α^+^) producing cells in the SL^+^ and IN^+^ groups, whilst a higher proportion of triple (IL-2^+^TNF-α^+^IFN-γ^+^) producers were observed in the IM^+^ group. Similarly, we have previously found that IM immunisation with the virosomal vaccine in combination with Matrix M™ adjuvant elicits a higher proportion of triple cytokine producing cells than IN vaccination [30] and that these cells are functionally superior to single and double cytokine producers [37]. Nevertheless, it has previously been proposed that IL-2^+^TNF-α^+^ CD4^+^ T cells have a higher long-term memory potential than IL-2^+^TNF-α^+^IFN-γ^+^ CD4^+^ T cells as IFN-γ producing cells represent a highly differentiated CD4^+^ T cell phenotype with a poor memory potential [64], [65]. Therefore, the optimal CD4^+^ T cell polarisation in terms of influenza vaccination needs to be further investigated.

Previous studies have found that whilst IN immunisation can redirect vaccine components to the olfactory bulbs and brain [14], [15], this is not a problem with the SL route [15] and this makes SL vaccination a potentially safer alternative than IN vaccination with regard to neurological side-effects. Nevertheless, we need to highlight that the only IN adjuvants associated with Bell's palsy has been A–B moiety toxins and their derivatives. In this context, it is well known that neurons display the specific receptors for the B subunit of these toxins and that these could facilitate retrograde homing to the CNS. Therefore, IN administration of adjuvants other than A–B moiety toxin derivatives may not have the same potential risk. However, the association between IN vaccination and neurological side effects may cause a scepticism against IN vaccines in the general population and its important to conduct extensive toxicology testing prior to the approval of any adjuvanted IN human vaccine. In this context, we show that the SL route is a promising alternative approach for the delivery of vaccines against potentially pandemic influenza strains. Particularly, the c-di-GMP adjuvanted H5N1 virosomes induces potent local and systemic immune responses when administered by the SL route. Therefore, we suggest further evaluation of the efficacy of this vaccine candidate in pre-clinical ferret studies.

## Supporting Information

Figure S1 **The heterosubtypic local and systemic antibody responses towards the pandemic H1N1 2009 virus.** Cross-reactive antibodies towards the pandemic H1N1 2009 virus (pH1N1) at two weeks after the second dose were measured by ELISA. **a**) The concentrations of cross-reactive IgA antibodies towards the pH1N1 virus in the nasal washes. **b**) The serum pH1N1-specific IgG antibody concentrations. Each bar represents mean antibody concentration+SEM. *** indicates statistically significant differences between groups (p<0.001, One-way ANOVA with Bonferroni's correction for multiple group comparison). Groups of six mice were vaccinated intramuscularly (IM), sublingually (SL) or intranasally (IN) with a virosomal H5N1 vaccine (NIBRG-14) with (+) or without (−) c-di-GMP adjuvant. An additional group received a mock vaccine (C) of c-di-GMP alone administered IN.
(EPS)

Figure S2 **The c-di-GMP adjuvanted vaccine induces a proliferative response irrespective of administration route and the highest H5N1-specific memory B-cell frequencies when administered mucosally.** The frequency of H5N1-specific memory B-cells (a) was measured by ELISPOT and the proliferative response (b) was measured in splenocytes 21 days after the second dose by stimulation with H5N1 virosomes. Each bar represents mean antibody concentration+SEM. * and ** indicate statistically significant differences between groups (p<0.05 and p<0.01 respectively, One-way ANOVA with Bonferroni's correction for multiple group comparison). Groups of six mice were vaccinated intramuscularly (IM), sublingually (SL) or intranasally (IN) with a virosomal H5N1 vaccine (NIBRG-14) with (+) or without (−) c-di-GMP adjuvant. An additional group received a mock vaccine (C) of c-di-GMP alone administered IN.
(EPS)
